# Case of Stone in the Bladder, with Operation of Lithotomy

**Published:** 1845-07-01

**Authors:** Alden S. Sprague


### Case of Stone in the Bladder, with Operation of Lithotomy.

Communicated for the Buffalo Medical Journal, by ALDEN S. SPRAGUE, M. D.

I was called about the first of April last, to see the son of George Stablie, now of this city, but a few years since from France, and found him suffering from some disease of the bladder. I learned from his parents that he was born in Paris, in the year 1834; that he was sent into the country to be nursed, under the care of a woman having charge of a vineyard, who was in the habit daily of drinking large quantities of Burgundy wine, and of giving it plentifully to the child.

He was restored to his parents at the age of 28 months, tolerably healthy, but in the habit of urinating in bed almost every night. Incontinence of urine, and a habit of constantly pulling at the prepuce, were the only circumstances which attracted attention until about five years since, when his parents first discovered that he manifested pain in making water. Since that time the pain has been constant, and latterly very severe.

At the time I was called, he presented the following appearances: age eleven years; stature not larger than an ordinary child at seven; countenance pale, emaciated; digestive organs impaired; incontinence of urine; severe pains in the region of the bladder, particularly immediately after evacuating it; frequent discharge of bloody mucus, accompanied by general debility.

Suspecting stone, I sounded him at my second visit, and found one.

After a consultation with my medical friends, an operation for the removal of the difficulty was proposed to the boy's parents, and their assent readily obtained. He was immediately put on a preparatory treatment, and the operation performed on the 22d of May, under tolerably favorable circumstances.

After the bowels had been evacuated by an injection, the staff was introduced. The stone being recognized by several medical gentlemen present, he was laid upon the table, and secured in the usual manner. I then commenced my first incision just below the scrotum at the raphe, and carried it to midway between the anus and tuberosity of the ischium; a few subsequent touches with the knife, aided by my finger nail, brought me to the membranous part of the urethra, when the groove of the staff was easily discovered. The urethra was severed to the extent of about half an inch. The staff was now seized with my left hand, and taking the gorget with the other, its beak was placed cautiously into the groove of the staff, balanced, then pushed steadily into the bladder. At this instant the boy made a violent struggle, the rectum became inflated, and was slightly wounded. Immediately after the incisions were completed, the finger was passed into the bladder, and the stone distinctly felt. I took a small forceps, introduced it on the withdrawal of my finger, seized the stone, and extracted it without difficulty.

The bladder was thoroughly rinsed with warm milk and water, a gum elastic tube inserted into the wound, the limbs fastened together, and the boy laid on his left side in bed. No vessel requiring a ligature was cut during the operation, nor was there any hemorrhage afterward demanding attention. Directions were given to have all motion avoided, and strict injunction to keep the patient entirely on barley or rice water and mucilages. The tube was removed on the second day. The urine passed partially on the tenth, and entirely on the fifteenth day. Liquid feces likewise passed through the wound for a short time. After the fifteenth day, however, all the discharges were passed naturally, and on the twenty-second day after the operation, the wound was entirely healed.

Two or three things naturally suggest themselves for consideration in the foregoing case. One is, that no case of stone in the bladder, as I am informed, has ever been known to have originated in this vicinity.

Another is, the manner in which this child was treated during infancy.

And still another, the readiness with which the parts healed, after the rectum had been wounded.

The stone was an inch in length, three fourths of an inch wide, and half an inch in thickness. It was of a reddish brown color, and probably belongs to the class of lithates.

*Buffalo*, *June* 20, 1845.

